# Beyond the State of the Art: Novel Approaches for Thermal and Electrical Transport in Nanoscale Devices

**DOI:** 10.3390/e21080752

**Published:** 2019-08-02

**Authors:** Robert Biele, Roberto D’Agosta

**Affiliations:** 1 Institute for Materials Science and Max Bergmann Center of Biomaterials, TU Dresden, 01062 Dresden, Germany; 2 Nano-Bio Spectroscopy Group and European Theoretical Spectroscopy Facility (ETSF), Universidad del Pais Vasco CFM CSIC-UPV/EHU-MPC and DIPC, Av. Tolosa 72, 20018 San Sebastian, Spain; 3 IKERBASQUE, Basque Foundation for Science Maria Diaz de Haro 3, 6 Solairua, 48013 Bilbao, Spain

**Keywords:** electronic transport, thermal transport, strongly correlated systems, Landauer-Büttiker formalism, Boltzmann transport equation, time-dependent density functional theory, electron–phonon coupling

## Abstract

Almost any interaction between two physical entities can be described through the transfer of either charge, spin, momentum, or energy. Therefore, any theory able to describe these transport phenomena can shed light on a variety of physical, chemical, and biological effects, enriching our understanding of complex, yet fundamental, natural processes, e.g., catalysis or photosynthesis. In this review, we will discuss the standard workhorses for transport in nanoscale devices, namely Boltzmann’s equation and Landauer’s approach. We will emphasize their strengths, but also analyze their limits, proposing theories and models useful to go beyond the state of the art in the investigation of transport in nanoscale devices.

## 1. Introduction

Some of the most spectacular advancements in the description of nature have come from the observation that apparently diverse effects are in reality based on the same physical principles. One of these is the realization that whatever interaction between two entities happens through the exchange of some physical quantity, either momentum, energy, spin, or particles. It is, therefore, of paramount importance to be able to describe how this transfer happens at a nanoscopic level, since these principles are usually fundamental to understand more complex systems, such as materials for energy applications, biological systems, and so on [1,2,3,4]. For example, it is now clear that our current inability in reproducing solar energy conversion that takes place normally in plants is related to the difficulties in understanding how light is transformed into an electrical current inside a leaf, in particular how the carriers are split and then move away from the light-receptor centers. At the same time, one key point in this energy conversion process is how excess energy is dissipated. Nature has developed for the leaf feedback mechanisms avoiding it being burnt if storing too much energy—these self-regulating mechanisms are yet outside our understanding and only recently with the advancement of both theoretical and experimental methods have some breakthroughs have been made [5,6].

In this review, we will discuss some of the most common approaches of widespread use in the nanoscale transport community. Here, we assume that a “device” of size ranging from 1 to a few hundreds nm is connected to some macroscopic metallic leads that can induce electrical and energy transfer through the device. We will focus on two fundamental models to describe this transport: on the one hand we will discuss the Landauer’s formalism for quantum transport. This is valid when the flow crosses the device essentially without being scattered, i.e., in the so-called ballistic regime: an accurate description when the mean free path of the particles, i.e., the distance between two scattering events, is larger than the dimensions of the device. On the other hand, we will consider the Boltzmann’s equation for transport. Here, we assume particle are effectively described by a distribution function and they can be scattered by particle–particle interaction, impurities, or by the device. In this case, the mean free path must be small, otherwise the description through an out-of-equilibrium distribution function does not hold. Both theories can be equally applied to the problem of energy and electric current transport, i.e., to the description of the dynamics of electrons and phonons. The ability of treating on essentially equal footing electrons and phonons allows for a comprehensive description of the device maintaining somewhat a consistent level of accuracy. In general, the outcome of the theory are the transport coefficients [1,2,4]. They serve in describing how the device responds to external stimuli such as a thermal gradient or a bias voltage. We will therefore focus on the electrical conductance σ, the Seebeck’s coefficient *S*, and the electron and phonon thermal conductances $\kappa_{e}$ and $\kappa_{p}$, respectively.

After having introduced the fundamental models, we will discuss some of their uses, generalizations, and other methods applicable to the intermediate regime where the mean free path is comparable with the dimension of the device. In particular, we will discuss the role of electron–electron and electron–phonon coupling in modifying the transport coefficients. The review is organized as follows: Section 2 introduces Boltzmann’s equation and Landauer’s quantum transport formalisms. We will discuss their formulation using the most advanced electronic structure methods and discuss critically their advantages and limits. This section also allows us to introduce a consistent notation for the rest of the presentation; In Section 3 we will discuss some recent attempts to go beyond the standard approaches. We will explore, for example, the role of electron–phonon interaction and strong electron correlation in affecting the transport coefficients, and introduce theories and models that allow description of these effects efficiently while maintaining the strength of the general formalisms; Finally, Section 4 contains an outlook of some potential lines of further investigation.

## 2. Static Approaches: Semiclassical and Quantum Transport Approaches

The calculation of the transport coefficients of a nanoscale device requires an accurate description of both electron and phonon (or, whenever translational invariance is broken, vibrational) properties. This description is used into standard methodologies to evaluate the transport coefficients and afterwards, e.g., the figure-of-merit of thermoelectric energy conversion. In this review, we will describe two of these methods, namely the Boltzmann’s transport theory which can be seen as a semiclassical method since it is based on the evaluation of the distribution function through velocities and density of states of the device, and the Landauer’s approach to quantum transport, which on the other hand describes a ballistic particle transfer through scattering between states in the leads [1,2,4]. There are many Approaches, however, of high scientific significance due to their accurate description of particle–particle interaction that we will not discuss here. One of these is the rate equations formalism [7], which can be made extremely accurate and describe strongly correlated system, but its wild scaling with the number of states reduces its applicability to either simplified models or small systems.

### 2.1. Semiclassical Boltzmann Transport

To calculate macroscopic transport coefficients, such as electronic or thermal conductivity, one needs to analyze the microscopic processes happening when electrons are scattered by phonons or impurities in a metal or semiconductor. The idea is to treat the electronic excitations as particles and follow their motion over time. For example, an electron in a metal exposed to an electric field E gets accelerated to a velocity v, and over time gains the kinetic energy

(1) Δϵ=vEt.

However, the velocity of the electron cannot grow forever as the electron will be scattered sooner or later with impurities or by phonons, changing its direction and losing part of its kinetic energy. If we do not know the exact microscopic details of how this scattering happens, we might just consider a macroscopic relaxation time τ. This is the average time an electron gets accelerated by the electric field before being scattered, i.e., the time between electron–phonon transitions. After the collision takes place the particle starts again with its acceleration parallel to the electric field until further scattering. The averaged velocity (over time and many different scattering events) due to acceleration and scattering is responsible for the finite electrical conductivity, as the current density is $J=ne\bar{v}$, where *n* is the electron concentration and *e* its charge.

Let us now consider the conductivity of a metal or semiconductor in more details. An electron in a crystal occupies states according to its distribution function f(r,k,t), giving the probability of finding an electron at position r and time *t* with the crystal momentum k. At equilibrium, when no fields or temperature gradients are applied, this is given by Fermi function,

(2) $$f_{0}\left(k,\mu ,T\right)=\frac{1}{e^{\left(\epsilon_{n}\left(k\right)-\mu \right)/k_{B}T}+1},$$

where μ is the total chemical potential and $\epsilon_{n}\left(k\right)$ the energy of an electron in the n-th band with momentum k, $k_{B}$ the Boltzmann’s constant and *T* the temperature. However, if we now apply, for example, a potential bias, electrons will get excited and the actual distribution functions shifts to higher energies. The equal interplay between acceleration on the one hand and collisions and scattering on the other, will possibly set up a steady-state condition. When we switch off the electrical field again, the system relaxes back to its equilibrium state.

In 1872, Boltzmann laid down an equation for *f* connecting thermodynamics with non-equilibrium kinetics [8]. Although this was long before the birth of modern quantum mechanics, his transport theory consists of a probabilistic description for the one-particle distribution function f(r,k,t). This equation for out-of-equilibrium situations is called the semiclassical Boltzmann transport equation (BTE). Here, we will lay down the basic equation and see how to solve it for systems which are close to equilibrium, while in Section 2.2 we review a fully quantum-mechanical treatment for transport properties. As mentioned before, we will treat crystal electrons as semiclassical particles fulfilling the equations

(3) $$\begin{array}{ccc} \frac{dk}{dt} & = & \frac{1}{\hbar }F_{e}, \end{array}$$

(4) $$\begin{array}{ccc} v & = & \frac{1}{\hbar }\nabla_{k}\epsilon_{n}\left(k\right). \end{array}$$

Here, $F_{e}$ is the force acting on the electron and v its velocity. Considering the total change in time of the distribution function

(5) $$\frac{df(r,k,t)}{dt}=\frac{\partial f}{\partial t}+\frac{\partial r}{\partial t}\cdot \frac{\partial f}{\partial r}+\frac{\partial k}{\partial t}\frac{\partial f}{\partial k}=\frac{\partial f}{\partial t}+v\cdot \nabla_{r}f+\frac{1}{\hbar }F_{e}\cdot \nabla_{k}f,$$

and setting it equal to the scattering rate, we arrive at the BTE,

(6) $$\frac{\partial f}{\partial t}+v\cdot \nabla_{r}f+\frac{1}{\hbar }F_{e}\cdot \nabla_{k}f=\frac{\partial f}{\partial t}{|}_{\mathrm{scatt}}.$$

The BTE states that the electron distribution changes due to in- and out-going scattering events which together form $\frac{\partial f}{\partial t}{|}_{\mathrm{scatt}}$. Although the Boltzmann transport theory is probabilistic, it is still classical as in quantum mechanics one cannot specify the canonical variables p and r simultaneously, due to the Heisenberg uncertainty principle, ΔpΔx≥ℏ. The assumption here is that the uncertainty in space and momentum is small enough compared to the system size that the electrons can be treated as particles. The scattering term, $\frac{\partial f}{\partial t}{|}_{\mathrm{scatt}}$, is the most interesting and complicated part of the BTE which accounts for the change of the electron probability due to electron–electron or electron–phonon scattering events, which we can treat quantum mechanically.

Without taking care of the exact form of the underlying scattering mechanisms, the constant relaxation time approximation (CRTA)assumes that the system seeks to return to the equilibrium configuration $f_{0}$ in a time τ after being disturbed by external electric or temperature fields. In general, τ, known as relaxation time, should depend on the direction of the scattering events, energy, and on the exact scattering mechanisms. However, the easiest version of the CRTA assumes one constant momentum relaxation time for all modes, direction, and scattering processes and the collision term can be written in the form [9,10]

(7) $$\frac{\partial f(r,k,t)}{\partial t}{|}_{\mathrm{scatt}}≃-\frac{\delta f(r,k,t)}{\tau }$$

Here, the rate of change of f(r,k,t) due to collisions is assumed to be proportional to the deviation from the equilibrium distribution, $\delta f=f-f_{0}$. To better understand the meaning of the CTRA, we assume no electric field present (no $F_{e}$) and spatial uniformity (gradient with respect to r vanishes). Then the BTE, Equation (6), in the CRTA takes the form

(8) $$\frac{\partial f}{\partial t}=-\frac{\delta f}{\tau },$$

which has the familiar solution for the deviation from equilibrium

(9) $$\delta f\left(t\right)=\delta f\left(0\right)e^{-t/\tau }.$$

This means that the perturbed system relaxes with a typical timescale τ to its equilibrium distribution. The CRTA is a crude approximation that fails for certain systems as we will see later in this section.

When going beyond the simple CRTA and concentrating on the phonon-limited carrier mobilities in semiconductors, it is common to consider the electron–phonon scattering as the dominant mechanism. Furthermore, we adopt the notation for crystals in k-space, as such, the distribution function depends on the band index *n* and the momentum k, i.e., $f_{nk}$. Therefore, for time-independent and homogenous fields, the BTE in k-space reads

(10) $$\frac{\partial k}{\partial t}\frac{\partial f_{nk}}{\partial k}=\frac{\partial f_{nk}}{\partial t}{|}_{\mathrm{scatt}}.$$

Neglecting magnetic fields and considering only the presence of electric fields, the BTE for phonon-limited carrier mobilities is given by [11,12]

(11) $$\begin{array}{ccc} eE\frac{\partial f_{nk}}{\partial k} & = & \frac{\Omega }{{\left(2\pi \right)}^{2}\hbar }\sum_{m,v}\int dq{\left|g_{mnv}\left(k,q\right)\right|}^{2}\\ & & \times \{f_{nk}\left[1-f_{mk+q}\right]\left[\left(n_{qv}+1\right)\delta \left(\epsilon_{nk}-\epsilon_{mk+q}-\hbar \omega_{qv}\right)+n_{qv}\delta \left(\epsilon_{nk}-\epsilon_{mk+q}+\hbar \omega_{qv}\right)\right]\\ & & -f_{mk+q}\left[1-f_{nk}\right]\left[\left(n_{qv}+1\right)\delta \left(\epsilon_{nk}-\epsilon_{mk+q}+\hbar \omega_{qv}\right)+n_{qv}\delta \left(\epsilon_{nk}-\epsilon_{mk+q}-\hbar \omega_{qv}\right)\right]\}. \end{array}$$

The first two terms in the second line represent scattering of an electron from energy band *n* with momentum k into the state m,k+q by either the emission $\left(n_{qv}+1\right)$ or the absorption ($n_{qv}$) of a phonon with frequency $\omega_{qv}$. The factor $f_{nk}\left[1-f_{mk+q}\right]$ make sure that the initial electronic state is occupied while the final state is unoccupied. The last two terms represent the backscattering events from state m,k+q towards the state in the energy band *n* with momentum k. Here, Ω is the volume of the primitive unit cell, the δ-functions ensure energy conservation during the scattering process and $n_{qv}$ is the Bose-Einstein distribution function, giving the probability that a crystal phonon with moment q is present in branch *v*. The term $g_{mnv}\left(k,q\right)$ is the electron–phonon coupling matrix element, which is normally calculated within density functional perturbation theory [13,14,15]. Taking the derivative with respect to the i-component of the electric field $E_{i}$, and linearizing the equation around the equilibrium distribution $f^{0}$, we get a direct equation for $\partial_{E_{i}}f_{nk}$ [12]

(12) $$\begin{array}{ccc} \frac{\partial f_{nk}}{\partial E_{i}} & = & e\frac{\partial f_{nk}^{0}}{\partial \epsilon_{nk}}v_{nk,i}\tau_{nk}+\frac{\Omega \tau_{nk}}{{\left(2\pi \right)}^{2}\hbar }\sum_{m,v}\int dq\frac{\partial f_{mk+q}}{\partial E_{i}}{\left|g_{mnv}\left(k,q\right)\right|}^{2}\\ & & \times \left\{\left[1+n_{qv}-f_{nk}^{0}\right]\delta \left(\epsilon_{nk}-\epsilon_{mk+q}+\hbar \omega_{qk}\right)+\left[n_{qv}+f_{nk}^{0}\right]\delta \left(\epsilon_{nk}-\epsilon_{mk+q}-\hbar \omega_{qk}\right)\right\}, \end{array}$$

where the inverse of the electron energy relaxation time due to the electron–phonon interaction is

(13) $$\begin{array}{c} \tau_{nk}^{-1}\left(T,\mu \right)=\frac{\Omega }{{\left(2\pi \right)}^{2}\hbar }\sum_{m,v}\int dq{\left|g_{mnv}\left(k,q\right)\right|}^{2}\{\left[n_{vq}+f_{mk+q}^{0}\right]\delta \left(\epsilon_{nk}-\epsilon_{mk+q}+\hbar \omega_{qv}\right)\\ +\left[n_{vq}+1-f_{mk+q}^{0}\right]\delta \left(\epsilon_{nk}-\epsilon_{mk+q}-\hbar \omega_{qv}\right)\}. \end{array}$$

Note here that the Fermi distribution for the electrons is a function of both temperature and chemical potential, while the phonon distribution function depends on the temperature. Equation (12) is the linearized BTE (LBTE) for phonon-limited carrier transport and is valid for most semiconductors where the acceleration of the free carriers is smaller than the thermal energy, $eEv\tau \ll k_{B}T$. We would like to point out that in contrast to the CRTA, here the relaxation time depends on the energy and momentum of the electron. The LBTE needs to be solved self-consistently for the variation of the electron distribution function with respect to the applied field, therefore also called iterative BTE. By neglecting the integral part of the LBTE, one obtains an equation for the distribution function which can be solved directly,

(14) $$\frac{\partial f_{nk}}{\partial E_{i}}=e\frac{\partial f_{nk}^{0}}{\partial \epsilon_{nk}}v_{nk,i}\tau_{nk}$$

and is called the self-energy relaxation time approximation (SERTA). This is due to the analogy that the relaxation time τ in Equation (13) is related to the Fan-Migdal electron self-energy by τnk−1=2ImΣnkFM [13]. In the SERTA, the electron mobility takes the form

(15) $$\mu_{\alpha \beta }\left(T,\mu \right)=-\frac{e\Omega }{n_{e}{\left(2\pi \right)}^{3}}\sum_{n\in \mathrm{CB}}\int dk\frac{\partial f_{nk}^{0}\left(T,\mu \right)}{\partial \epsilon_{nk}}v_{nk,\alpha }v_{nk,\beta }\tau_{nk}\left(T,\mu \right).$$

When calculating the mobility within SERTA or LBTE, the relaxation time in Equation (13) is evaluated on a very fine k-grid by integrating over a dense q-grid for the phonons. This direct Brillouin zone sampling is computationally very demanding and hence cannot be applied for complex material screening. For example, the EPW code [16] calculates the electron–phonon coupling on a coarse grid in the BZ and maps it onto a fine grid by using Wannier’s function interpolation. This method is however still very costly and therefore other approaches are needed to tackle the calculations of thermoelectric transport properties. One approach solves the BTE within the CRTA, Equation (7): This leads to good results for electrical conductors where the energy relaxation time depends weakly on the electron energy, ϵ, [10,17] and allows for a single constant relaxation time. However, when performing the CRTA one needs to estimate the relaxation time within simplified models, such as the deformation potential (DP) approximation [18,19,20] or Allen’s formalism [21,22]. For most materials, τ is strongly anisotropic, depending on energy and carrier concentration and the CRTA cannot be applied and we need to revert to first-principles computations for predicting $\tau_{nk}$. The so-called electron–phonon averaged (EPA) approximation [23] turns the demanding integral over momentum in Equation (13) into an integration over energy. This is done by replacing quantities that depend on momentum ($|g_{mnv}{\left(k,q\right)|}^{2}$, $\omega_{vq}$) by their energy-dependent averages ($g_{v}^{2}\left(\epsilon_{nk},\epsilon_{mk+q}\right)$ , $\bar{\omega_{v}}$). This allows a much coarser grid in the electron energies therefore reducing computational cost drastically. Technical details and derivations can be found in [23] and the final relaxation time within the EPA is given by

(16) $$\begin{array}{c} \tau^{-1}\left(\epsilon ,\mu ,T\right)=\frac{2\pi \Omega }{2\hbar }\sum_{v}\{g_{v}^{2}\left(\epsilon ,\epsilon +\bar{\omega_{v}}\right)\left[n\left({\bar{\omega }}_{v},T\right)+f^{0}\left(\epsilon +{\bar{\omega }}_{v}\right)\right]\rho \left(\epsilon +{\bar{\omega }}_{v}\right)\\ +g_{v}^{2}\left(\epsilon ,\epsilon -\bar{\omega_{v}}\right)\left[n\left({\bar{\omega }}_{v},T\right)+1-f^{0}\left(\epsilon -{\bar{\omega }}_{v}\right)\right]\rho \left(\epsilon -{\bar{\omega }}_{v}\right)\}, \end{array}$$

where ${\bar{\omega }}_{v}$ is the average phonon mode energy and ρ(ϵ) is the electron density of states per unit energy and unit volume. The EPA is implemented in the Quantum Espresso suite [24] and Boltztrap code [25].

**Figure 1:**
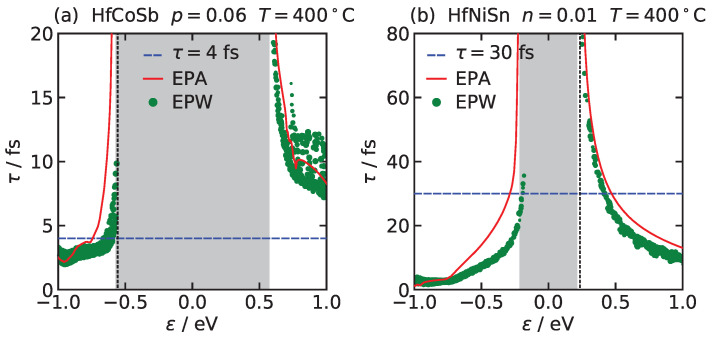
Relaxation time for (**a**) HfCoSb and (**b**) HfNiSn calculated within the EPA (Equation (16)) compared to the complete sampling of the Brillion zone (Equation (13)). Both approaches are in good agreement and the CRTA (dashed blue line) might fail to accurately calculate the conductivity. Reprinted with permission from [23]. Copyright (2018) John Wiley and Sons.

Figure 1 compares the relaxation time calculated within Equation (13) by the EPW code and within the EPA, Equation (16) for (a) HfCoSb and (b) HfNiSn. We see that the relaxation times in both approaches are in a good agreement, and furthermore that approximating the relaxation times with a constant, as done in CRTA, might fail for predicting the electron conductivity of real materials.

### 2.2. Landauer–Buttiker and Quantum Transport

The Landauer–Buttiker’s formalism (LB) is an elegant and economic way to study transport through molecular devices [26,27,28]. The idea at its core is deceivingly simple: The overall device is separated into three or more regions of space, one of which the central, usually characterizes the physical properties of the overall system. The other regions serve as sink or reservoir of electrons. The only property that is required from the reservoirs is that they connect smoothly with the central region, thus avoiding backscattering due to the contacts, and that they are either perfect emitters or absorbers, in the sense that one electron entering them cannot leave through the central region again. This requires also that their spectral density is essentially flat over a wide range of energies around the Fermi level of the central system. LB then describes the currents flowing between the leads, through the probability that an electron entering through the lead *i* is scattered by the central region, and thus leaves through the lead *j*, $T_{i,j}$. In this respect thus the LB formalism is a scattering theory and the transmission probability is its central ingredient. Finally, one sums over all the allowed electron energies [26,27,28]. The electrical current is therefore given by

(17) $$I_{i,j}=\frac{2e}{h}\int d\epsilon \left(f_{i}\left(\epsilon \right)-f_{j}\left(\epsilon \right)\right)T_{i,j}\left(\epsilon \right),$$

where $f_{i}$ is the Fermi function describing the electron occupations of the lead *i*, *e* is the electron charge, *h* is the Plank’s constant, and the factor 2 takes into account spin degeneracy. Generally, the Fermi functions depend upon the local temperature $T_{i}$ of the lead *i*, their chemical potential $\mu_{i}$, and the bias applied to the lead, $V_{i}$. Without loss of generality, in the presence of the bias $V_{i}$ we can assume that $\mu_{i}=0$. It is important to realize the important approximation of the model: we are assuming that the occupation of the electrons entering from the lead *i*, depends only on the equilibrium physical parameter of the *i* lead. Finally, we have also assumed that the central system is essentially one dimensional—in this case, the density of states and the electron velocity cancel out to achieve the universal result of Equation (17). A thorough discussion of the derivation and the physical implications of the Landauer transport theory goes way beyond the aim of this review and the topic has been discussed in a large number of publications, including reviews and monographs [1,2,4,26,27,28,29].

Given the current *I* from the different terminals, one can easily calculate the two-terminal conductance, in the linear-response regime $\delta V_{i,j}\to 0$,

(18) $$\sigma_{i,j}=\lim_{\delta V_{i,j}\to 0}\frac{I_{i,j}}{\delta V_{i,j}}=\frac{2e^{2}}{h}\int d\epsilon f^{\prime }\left(\epsilon \right)T_{i,j}\left(\epsilon \right).$$

where $f^{\prime }\left(\epsilon \right)$ is the first derivative of the Fermi function with respect to the energy, and $\delta V_{i,j}=V_{i}-V_{j}$ the bias difference between the leads *i* and *j*. The simplest most striking prediction that the LB theory makes is the quantization of the conductance. Indeed, in the low temperature limit, $f^{\prime }\to \delta \left(\epsilon -E_{F}\right)$ where $E_{F}$ is the Fermi energy of the leads, so that [26,27,28]

(19) $$\sigma_{i,j}=\frac{2e^{2}}{h}T_{i,j}\left(E_{F}\right).$$

If we assume that $N_{i,j}$ states are fully open to transport current, i.e., their transmission probability goes to 1, we have

(20) $$\sigma_{i,j}=\frac{2e^{2}}{h}N_{i,j}$$

as it has been carefully verified in many experiments, for example in the quantum Hall effect [30,31] or in the ballistic regime of a quantum point contact [32,33,34]. Equation (19) is what is normally called the “Landauer’s formula” and it is deceivingly simple. However, its physical interpretation has puzzled the community for many years. In particular, one question that arises naturally is from where this conductance originates. Clearly, we are describing an almost ideal system. The electrons flow seamlessly from the leads to the central region, and they are transmitted to the other lead with probability 1, so there is no scattering in these states. In addition, yet, we have a finite conductance thus associated with energy loss and potential drops. The solution of this apparent paradox lies in the different dimensionality of the leads with respect to the central system. The “adjustment” of the wave-function to adapt to the reduced dimensionality of the center causes a charge accumulation at the interfaces between the center and the leads, no matter how smooth these interfaces are. This extra charge that accumulates as soon as we contact the central region, creates a finite bias that opposes the one applied to the reservoir and finite conductance appears.

One of the main advantages of the Landauer’s formalism lies in treating on the same footing different physical problems. For example, the study of thermal transport both by electrons and phonons can be easily recast in the form of a scattering problem for the electrons and phonons trough the central region. Therefore, the energy current due to the electrons is evaluated as

(21) $$J_{i,j}^{e}=\frac{2}{h}\int d\epsilon \left(\epsilon -\mu \right)\left(f_{i}\left(\epsilon \right)-f_{j}\left(\epsilon \right)\right)T_{i,j}\left(\epsilon \right),$$

while the phonon contribution is given by

(22) $$J_{i,j}^{p}=\frac{\hbar }{2\pi }\int d\omega \omega \left(n_{i}\left(\omega \right)-n_{j}\left(\omega \right)\right)T_{i,j}\left(\omega \right),$$

where $n_{i}$ is the Bose distribution of the phonons in the lead *i* kept at temperature $T_{i}$. In the following we assume that $T_{i}=T+\Delta T_{i}$, where $\Delta T_{i}$ defines the difference between the temperature of the lead *i* and the reference temperature *T*. As we did for the electrical conductance, we can calculate the thermal conductance for the phonons and the electrons by simply assuming that a small thermal gradient is present between the leads. Moreover, if one assumes that both the potential and thermal gradient are small, the Landauer formalism reduces to the Onsager linear-response out-of-equilibrium transport theory, with the advantage of providing a reliable way of calculating the Onsager’s coefficients [1,35]. Indeed, if one defines the functions (Lorentz’s integrals)

(23) $$L_{n}=\int d\epsilon {\left(\epsilon -\mu \right)}^{n}f^{\prime }\left(\epsilon \right)T_{i,j}\left(\epsilon \right)$$

we have the Onsager’s relations (restricting ourselves to only two leads)

(24) $$\begin{array}{ccc} I & = & \frac{2e^{2}}{h}L_{0}\Delta V-\frac{2e}{hT}L_{1}\Delta T, \end{array}$$

(25) $$\begin{array}{ccc} J_{e} & = & -\frac{2e}{h}L_{1}\Delta V+\frac{2}{hT}L_{2}\Delta T. \end{array}$$

The electron transport coefficients, namely the electrical conductance, the thermal conductance and the Seebeck’s coefficient then follow from their physical definitions and are expressed solely in terms of the functions $L_{n}$. Indeed, the electrical conductance is ${\sigma =I/\Delta V|}_{\Delta T\equiv 0}=2e^{2}L_{0}/h$, the Seebeck’s coefficient is ${S=-\Delta V/\Delta T|}_{I=0}=-L_{1}/eTL_{0}$, while the thermal conductance is ${\kappa =J/\Delta T|}_{I=0}=2\left(L_{2}-L_{1}^{2}/L_{0}\right)/hT$. Notice that the physical definition of the transport coefficients is in general not restricted to the linear-response regime, so one could define the Seebeck coefficient as S=−ΔV/ΔT also for large ΔT, but the expression in terms of the Onsager’s coefficients and Lorentz integrals is valid only in linear response.

Moreover, the formula for the phonon energy current Equation (22) predicts the surprising result that the low energy phonons, usually responsible for large part of the energy transport since they have the longer wavelength, have a quantized thermal conductance,

(26) $$\kappa_{p}=\frac{k_{B}^{2}\pi^{2}}{3h}T,$$

per each open channel, as predicted by Rego and Kirczenow [36] and verified by Schwab et al. [37].

Although the Landauer’s theory appears quite natural, its physical implications are far reaching as we have seen with the introduction of the quantum of conductance [30,31,32,33,34] and the quantum of thermal conductance that has been observed [37]. However, the maximum strength of the theory is reached when it is coupled with the standard method for electronic structure calculations, namely static Density Functional Theory (DFT), and the non-equilibrium Green’s function formalism (NEGF). These two methods made the Landauer’s theory and the Boltzmann’s transport theory, the base for almost any recent transport calculations. DFT indeed produces a reliable description of the system energy and electron density and of the electronic band structure, both necessary for the evaluation of the transmission probability although its use on transport modeling should be taken with care as we will discuss shortly. Starting from the DFT description the NEGF method can express the transmission coefficients $T_{i,j}$ in terms of the Green’s functions of the leads and those of the central system. This combination allows for an almost parameter-free description of quantum transport at the atomistic level and it is the actual reference method for this kind of physical problems.

So far we have not discussed how to calculate the transmission probabilities between the leads *i* and *j*, $T_{i,j}$. This is usually a difficult problem since to make an accurate description of the scattering process we need modeling the states of the electrons inside the central region. Fortunately, we can combine the predictive power of DFT, which precisely describes the states of the system with the NEGF formalism which (as we will see) allows calculation of the transmission probability. A detailed derivation of all the results would be outside the scope of this review. There are a significant number of detailed monographs dedicated to the subject and we refer the interested reader to them [29,38]. Given a Hamiltonian *H*, we formally define its advanced and retarded Green’s functions through (we set from now on ℏ=1)

(27) $$\left[-i\frac{d}{dt}-H\right]G^{R(A)}\left(t,t^{\prime }\right)=\delta \left(t-t^{\prime }\right),$$

with the conditions $G^{R}\left(t,t^{\prime }\right)=0$ if $t<t^{\prime }$ and $G^{A}\left(t,t^{\prime }\right)=0$ is $t>t^{\prime }$. An equivalent definition of the retarded and advanced Green’s functions is based on their representation in terms of energy, i.e., after a Fourier transform,

(28) $$\left[(E\pm i\eta )-H\right]G^{R(A)}\left(E\right)=1$$

where η is an infinitesimal positive quantity that serves to establish the analytical properties of $G^{R(A)}$. When we select a basis set for the Hilbert space, the Green’s functions (as well as the Hamiltonian) can be represented as infinite matrices. However, normally only a certain number of states will be relevant for the dynamics (in our case those close to the Fermi energy or electrochemical potential μ) and therefore only a submatrix of the total Green’s function will be needed.

**Figure 2:**
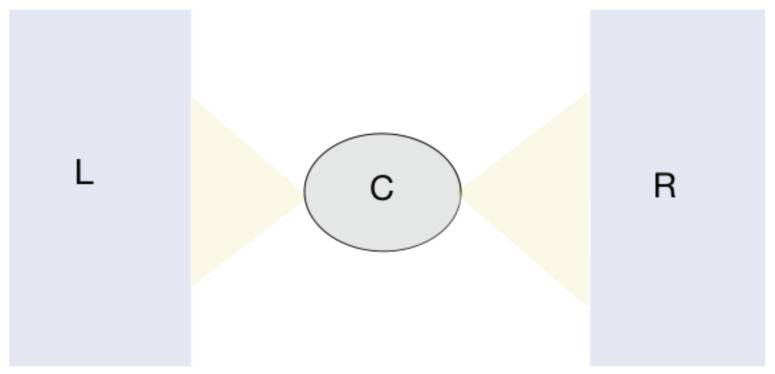
A central region (C) is connected to two external energy and particle left and right reservoirs (L and R) by metallic contacts. Current flows between the reservoirs when a temperature or bias gradient is established. The reservoirs are semi-infinite, namely the left proceeds from −∞ to C, the right goes from C to +∞.

For the study of electron transport through a nanoscale device, we need to describe the system (a central region) coupled to at least two external reservoirs. A pictorial representation of a device is given in Figure 2.

If separated, each of these objects can be described by their Hamiltonian and Green’s functions. When we couple the central region with the reservoirs, we are effectively introducing an interaction potential that couples states in the reservoirs with states in the leads. We introduce the following notation: $H_{A}$ with A=L,R, or *C* is the Hamiltonian of the left or right reservoir, or the central region, respectively; $V_{AB}$ with AB=RC,LC,CR, or RL represent the coupling between the central region with the left and right reservoirs, respectively. $V_{AB}$ can be for example a tunneling Hamiltonian between the lead and the central system. We assume there is not direct coupling between the reservoirs, so electrons must travel through the central region. In a matrix representation, we can think of the total Hamiltonian as a matrix whose sub-matrices are $H_{A}$ and $V_{AB}$,

(29) $$H=\left(\begin{array}{ccc} H_{L} & V_{LC} & 0\\ V_{CL} & H_{C} & V_{CR}\\ 0 & V_{RC} & H_{R} \end{array}\right).$$

Clearly, one could define a Green’s function associated with this Hamiltonian whose dynamical equation can be written as

(30) $$\left(\begin{array}{ccc} E-H_{L}+i\eta & -V_{LC} & 0\\ -V_{CL} & E-H_{C}+i\eta & -V_{CR}\\ 0 & -V_{RC} & E-H_{R}+i\eta \end{array}\right)\left(\begin{array}{ccc} G_{L}^{R} & G_{LC}^{R} & 0\\ G_{CL}^{R} & G_{C}^{R} & G_{CR}^{R}\\ 0 & G_{RC}^{R} & G_{R}^{R} \end{array}\right)=\hat{1},$$

where we have used the previous notation for the elements of $G^{R}$, and $\hat{1}$ is the identity matrix. This system of equations can be solved exactly to first express $G_{LC}^{R}$ and $G_{RC}^{R}$ in terms of $G_{C}^{R}$ and then, solve for the latter. The exact result is

(31) $$G_{C}^{R}={\left[E+i\eta -H_{C}-\Sigma^{R}\right]}^{-1}={\left[E-H_{C}-\Sigma^{R}\right]}^{-1}$$

where we have defined the self-energy

(32) $$\Sigma^{R}=V_{LC}^{†}{\left(E+i\eta -H_{L}\right)}^{-1}V_{LC}+V_{RC}^{†}{\left(E+i\eta -H_{R}\right)}^{-1}V_{RC},$$

where with a † we indicate the Hermitian conjugate of *V*. Notice that in $G_{C}^{R}$ the analytic properties are finally determined by those of the self-energy Σ and one can therefore neglect the terms iη. A similar equation can be derived for $G_{C}^{A}$. It is important to point out that in this theory, the leads enter both in the interaction with the central region through $V_{LC}$ and $V_{RC}$ and their isolated Green’s function $G_{L(R)}^{R}={\left(E+i\eta -H_{L(R)}\right)}^{-1}$.

To solve this set of equations, one assumes that there is no particle–particle interaction in the leads. This approximation can be justified by observing that they are thought of as normal metals and thus the screening length is relatively small, thus particles can be treated as weakly interacting if not independent. Within this scheme, $G_{L(R)}$ are uniquely determined and can be used to arrive at the lead self-energies to solve for $G_{C}^{R}$ and $G_{C}^{A}$. In these last quantities, however, we cannot neglect particle–particle interaction. Due to the complexity of the problem, the exact many-body Hamiltonian $H_{C}$ is replaced with the so-called Kohn–Sham (KS) Hamiltonian, where an external single-particle potential (generally unknown) serves to mimic the effect of the interaction (see also the following sections for somewhat deeper discussion) [39,40,41,42]. The KS Hamiltonian is tailored to reproduce the exact ground-state energy and density, but it often produces an accurate description of the total Green’s function of the isolated central region.

Finally, we connect the Green’s function formalism with the Landauer’s approach to quantum transport, since the former gives direct access to the transmission probability $T_{i,j}$. This step can be done by, for example, introducing the states for the left and right leads and solve the scattering problem with the central region by using the Green’s functions. After some manipulations, one writes the transmission function T(E) as [43]

(33) $$T\left(E\right)=\mathrm{Tr}\left(\Gamma_{L}G_{C}^{A}\Gamma_{R}G_{C}^{R}\right),$$

where $\Gamma_{L(R)}=i\left(\Sigma_{L(R)}^{R}-\Sigma_{L(R)}^{A}\right)$ is the so-called spectral function of the leads. Other approaches or models to the calculation of the transmission probability are clearly possible, and provide the tools to investigate novel and interesting phenomena, see for example Ref. [44] and the references in that focus issue.

This formalism is suited to describe both thermal and electrical transport since we have never specified the kind of gradient we maintain between the reservoirs. We are therefore entitled to consider both a bias voltage, and a temperature gradient that modifies the particle distribution functions in the reservoirs.

Quite naturally the formalism can be extended to consider the transport of energy through phonons or more generally vibrations. Formally, the only difference lies in replacing the particle Green’s function *G* with the vibration Green’s function *D* which is a solution of

(34) $$\left(\omega^{2}+i\eta -H_{v}\right)D^{R}\left(\omega \right)=1$$

where ω is the frequency of the vibration, and $H_{v}$ the Hamiltonian function describing the vibration dynamics. Following the same steps as before, one can introduce the transmission function of the vibration T(ω) and derive a formula similar to Equation (33).

It is fair to examine here some of the problems one might face when using DFT + NEGF for the electronic transport calculations:

- The original static DFT is in principle limited to provide the ground-state density and energy and nothing about the excited states of a system. Whatever result one extracts from the theory for example on the band-gap, band structure (and related quantities) should then be checked with independent methods and with experiments. It is well known for example that DFT with its standard approximation tends to underestimate the band-gap of many materials.
- Although the use of the NEGF greatly extends the applicability of the theory, the approximations behind the standard DFT codes makes the results not suitable for strong correlated materials where the effect of the Coulomb interaction is stronger. We will come back on this point later.
- Even if the description of the excited states happened to be accurate, the electron and heat current are dynamical quantities so in principle beyond the static approach of DFT. There are methods to go beyond this limit while remaining in the realm of a time-independent formalism. “Dynamical corrections” need to be included in the theory to take dynamics into account [45,46].

## 3. Advanced Methods

The Boltzmann’s equation and the Landauer-Büttiker formalism can be considered the de-facto standard methods to study transport in nanoscale devices, especially when coupled with accurate electronic structure calculations of the Density Functional Theory. It has become clear however that these methods have severe limitations that reduce the ensemble of systems to which they can be applied. In this section, we will discuss some attempts we made to go beyond and include novel effects outside the limitations of the state of the art. Some of these methods are admittedly in their infancy and a consistent amount of work is necessary before they can be admitted in the mainstream research methods. We hope that this section can motivate the interested readers to enter this enticing field which still bears endless possibilities.

### 3.1. Time-Dependent Density Functional Theory

Density Functional Theory proved an important point of quantum mechanics, i.e., that some exact properties of a many-body interacting system can be extracted from the study of a Doppelgänger of non-interacting particles [40,41,42,47]. The simplification brought about cannot be underestimated: it is enough to think that an interacting system requires an exponentially large Hilbert’s space for its accurate numerical description, while the same system of non-interacting particles requires only a polynomially large (with the number of states) space. This difference separates being able to treat just a few particles from studying complex molecules or crystal unit cells. There are two prices to pay for this simplification; On the one hand, we are ready to give up the complete information about the many-body system and study only certain quantities. The standard DFT formalism was aimed at the ground-state energy and density. Any other quantity that is then obtained in DFT must then be checked against other models; On the other hand, the particle–particle interaction is *replaced* in the so-called Kohn-Sham (KS) system with a non-linear external potential (known as KS potential) that is assumed to depend only on the single-particle ground-state density [39,47,48]. This potential is clearly unknown and we need to revert to some sort of approximation to make the theory useful. Standard approximations have been developed and admittedly work rather well especially when dealing with atoms and small molecules, but failure is also around the corner. Indeed, some of the standard problems of DFT in dealing with transport calculation derives from a combination of these points: band-gap and transport coefficients are normally not static ground-state properties, and the standard approximations for the KS potential consistently underestimate the electronic band-gap. Finally, there are classes of problems that are outside the realm of standard DFT, such as for example the calculation of spectroscopic quantities. A detailed introduction to DFT would bring us far away from the scope of this review. We can however, recommend excellent introductory material for the interested reader [40,41,42,47].

There are many ways to go beyond these issues. We can formulate the theory in the time domain, in such a way that we can extract the exact time dynamics of some of the quantities of the many-body interacting system [49,50,51]. This is the case of Time-Dependent Density Functional Theory. Meanwhile, we can improve the standard approximations of the KS potential, for example introducing corrections that take into account some part of the strong correlation between particles in certain regimes [52].

In the next sections we will introduce the Time-Dependent Density Functional Theory (TDDFT) [40,41] and its further extensions and later the so-called “i-DFT” that can be used to introduce strong correlation effects, such as Coulomb blockade into a TDDFT formulation (see Section 3.2) [53,54].

#### 3.1.1. Time-Dependent Density Functional Theory—Fundamentals

The standard approach of DFT is based on the existence of a one-to-one mapping between the exact ground-state density and the external potential applied to a quantum system [48]. Furthermore, one can extend this mapping between two systems, the many-body interacting “real” system and a many-body non-interacting “Kohn-Sham” system [39] where we introduce an effective external potential (dubbed KS potential). This second mapping allows for calculating quantities, i.e., the ground-state density and energy, belonging to the real system by using the KS system, bringing about a large numerical simplification. DFT is therefore the actual method of choice for calculating electronic structure of materials as well as the energies of complex atoms and molecules [42]. The KS mapping was later extended to the dynamics of the single-particle density by Runge and Gross [55]. The existence of this second mapping lays the foundations for performing, e.g., numerical spectroscopy with so-called ab-initio methods, i.e., without—in principle—any fitting parameter [41]. TDDFT can theoretically be used for studying electrical transport (see for example Ref. [56,57,58,59,60]), but the information it can provide is only partial [61,62].

It appears natural therefore, from the Runge and Gross’ theorem (RG), to establish a mapping between the single-particle current density and the external (vector) potential applied to the real system [63]. In principle, this Time-Dependent Current-Density Functional Theory (TDCDFT) should be the workhorse for charge transport calculations, but the lack of suitable approximations for the KS’s potential renders the theory of little use at the moment. One the other hand, addressing thermal transport phenomena within DFT would require instead a non-equilibrium theory based on either a local temperature or a local energy density. Recently, a functional theory based on the excess energy density as the basic variable has been presented [64,65] which is suited to study thermoelectrical phenomena in the static and time-dependent case. In the following, we will look only at standard TDDFT.

The RG theorem proves that for a general time-dependent Hamiltonian *H* that describes the dynamics of a many-body system,

(35) $$H\left(t\right)=T+V_{\mathrm{int}}+V_{\mathrm{ext}}\left(t\right)$$

there exists a one-to-one mapping between the single-particle density n(r,t) and the external potential $V_{\mathrm{ext}}\left(r,t\right)$, given the initial conditions. Here, *T* is the kinetic energy, $V_{\mathrm{int}}$ the particle-particle interaction, and $V_{\mathrm{ext}}$ the time-dependent external potential. The proof of the theorem assumes that both density and potential can be expanded in series around the initial time: a detailed proof and the physical assumption on which this is based can be found in [40,41]. It has since been shown that there exists a system of non-interacting particles whose single-particle density is identical at each time to n(r,t) of the interacting system [66]. The existence of this mapping then allows investigation of the dynamics of a system by looking at its non-interacting Doppelgänger. Clearly, this brought about the same reduction of computational requirements as the standard DFT. However, as was the case with DFT, we are exchanging some of the physical information about the system to reduce the dimensionality of the problem. It should then come as no-surprise that TDDFT cannot for example reproduce the exact single-particle current density j(r,t) since some of its contributions are not derivable from the knowledge of the density alone [67]. Indeed, if starting from the continuity equation,

(36) $$\partial_{t}n\left(r,t\right)=-\nabla \cdot j\left(r,t\right),$$

we need to conclude that given the density we can determine only the longitudinal part of j(r,t) and any term $j_{T}$ such that $j\left(r,t\right)=j_{L}\left(r,t\right)+j_{T}\left(r,t\right)$ and $\nabla \cdot j_{T}\equiv 0$ cannot be obtained from Equation (36). However, some information about the total current can still be obtained from the continuity equation [68].

As with the static DFT, we study the KS Hamiltonian $H_{\mathrm{KS}}$,

(37) $$H_{\mathrm{KS}}=T_{S}+V_{\mathrm{KS}}$$

where $T_{S}=\sum_{i}p_{i}^{2}/2m$ is the kinetic energy of the non-interacting particles of mass *m* and momenta $\left\{p_{i}\right\}$, and $V_{KS}=V_{\mathrm{ext}}+V_{\mathrm{Hxc}}$. The potential $V_{\mathrm{Hxc}}$ is the sum of the Hartree and exchange–correlation (xc) potentials that replace the particle–particle interaction. By the RG theorem $V_{\mathrm{Hxc}}$ is a functional of the density only, $V_{\mathrm{Hxc}}=V_{\mathrm{Hxc}}\left[n\right]\left(r,t\right)$, and the dynamics of the single-particle density of the real system evolving with Hamiltonian *H* is identical to the dynamics of the single-particle density evolving with $H_{\mathrm{KS}}$. Notice that in principle, given the particle–particle interaction of the original many-body problem, $V_{\mathrm{Hxc}}$ is a universal functional and does not depend on the external potential $V_{\mathrm{ext}}$. This implies for example that if we are interested in the dynamics of an electronic system, $V_{\mathrm{Hxc}}$ is the same either if we are studying an atom, a molecule, a slab, or a bulk, i.e., this potential is transferrable to whatever system we want to study. This high transferability makes finding reliable approximation for $V_{\mathrm{Hxc}}$ difficult. Indeed, the universal $V_{\mathrm{Hxc}}$ for the electrons contains the physical information needed to go from the standard weakly interacting Landau’s Fermi liquid, to the strongly correlated Wigner’s crystal or superconducting phases. Notice that for the electrons, a large part of $V_{\mathrm{Hxc}}$ is given by the Hartree’s (mean-field) interaction, and only a relatively small contribution of the overall energy determines all these interesting phases of matter. Therefore, standard approaches to build some approximation to $V_{\mathrm{Hxc}}$ are based on interpolating the numerical solution of the many-body problem with some known high- and low-density limits [69,70]. Many of these approximations are static. To apply them to the dynamics, a common method is the so-called adiabatic local density approximation (ALDA). In this approximation, we take a static $V_{\mathrm{Hxc}}\left[n\right]$ and replace the static density with the instantaneous density n(r,t). Indeed, this neglects the history of the system—while we generally expect that the xc potential to be history dependent. For example, one can show that in this approximation there is not relaxation induced by particle–particle interaction, in contrast with observation [71,72].

### 3.2. Strongly Correlated System

The Landauer approach coupled with Density Functional Theory through the Non-Equilibrium Green’s function formalism provides a reliable and presently standard method to calculate the transport properties of many devices. Countless are the successes of the theory, far beyond its actual range of applicability. Nonetheless there are many reasons to go beyond this state of the art. A simple reason is that we need alternative methods to provide benchmarks for the DFT + LB theory and learn from them. Indeed, the strength of DFT lies also in adapting the KS potential to the cases under investigation. On the other hand, we might need to go beyond some of the fundamental approximations and thus build a different theory.

Meanwhile, a striking example of the limits of the DFT + LB theory is related to one of the most fascinating outcomes of the so-called mesoscopic physics. Imagine that the device we place between two leads is a small molecule or a quantum dot. Both these systems are thought of having just a few states close to the Fermi energy (or the electrochemical potential). Imagine that one electron enters the device and occupies the lowest energy state. The next electron then faces an increased energy barrier, since beside the energy to occupy the lowest available energy states it also musts overcome the Coulomb interaction with the other electron. Normally, for large devices this energy is small since the electron density is “diluted”, but when we consider small dots or molecules, the Coulomb interaction might be the dominating energy scale and transport can be “blocked” until either the first electron leaves the device or the second electron has enough energy to overcome the Coulomb interaction. A standard DFT + LB approach to this problem is most likely going to fail. Indeed, DFT describes the electrons through their density and therefore it does not produce the sharp energy transition due to the addition of a single electron. This effect goes normally under the name of “Coulomb blockade”, and it is the epitome of a strongly correlated system where essentially the dynamics is dictated by electron interaction and correlation.

However, it is important to point out that these limits are related to our ability of inventing KS potentials able to describe certain physical regime. Per se, DFT can describe the ground-state properties of the system and thus give the exact energy for the single and double occupied electron states. It is our inability of encoding these effects into the KS potential that makes the theory fails. Indeed, progress has been made to include strong correlation into the KS potential into a pure DFT scheme. Here, we will consider the extension of DFT to deal with the Coulomb blockade regime. To do that, we first need to extend the theory to include the transport properties in a more accurate way. As we will see this step corrects the electronic conductance that is calculated from the standard DFT approach.

Our starting point is the observation that generally speaking, the quantities we want to investigate in studying the device of Figure 2 are the local electron density n(r) and the total current *I* flowing from one reservoir to the other due to a thermal gradient or a bias voltage. For the moment, we will focus on the steady state, i.e., we assume the system has evolved from an initial state and approached, as time goes by, a constant density and current. The external fields that we are applying are the bias voltage *V* and the gate voltage *v* which controls the electron density and the total number of particles. We assume that the nuclear potential is not affected by the electron distribution and it is, therefore, constant (we assume uniform temperature). We see *V* and *v* as a perturbation and n(r) and *I* as response. To make a DFT theory for this set of variables, we need to prove that they are uniquely connected. Specifically, that the pair n(r) and *I* uniquely determine both *V* and *v*. Moreover, we are interested in n(r) and *I* only inside a finite region, R, surrounding the device. The proof of the theorem entails the evaluation of the Jacobian of the mapping $\left\{n(r),I\right\}\to \left\{v(r),V\right\}$ for any r∈R. One can prove that around V=0 this mapping is invertible since the Jacobian does not vanish [53]. We can therefore follow the KS construction and find a system of non-interacting particles which can reproduce n(r) and *I* of the original system by replacing the interaction with the external potentials

(38) $$\begin{array}{ccc} V_{S}\left[n,I\right] & = & V\left[n,I\right]+V_{xc}\left[n,I\right] \end{array}$$

(39) $$\begin{array}{ccc} v_{S}\left[n,I\right] & = & v\left[n,I\right]+v_{Hxc}\left[n,I\right] \end{array}$$

where $V_{xc}$ and $v_{Hxc}$ are the xc potentials (in $v_{Hxc}$ we include also the Hartree mean-field potential). n(r) and *I* in the KS system are determined by

(40) $$\begin{array}{ccc} n(r) & = & 2\int \frac{d\epsilon }{2\pi }\left[f\left(\epsilon -\frac{V+V_{xc}}{2}\right)A_{L}\left(r,\epsilon \right)+f\left(\epsilon +\frac{V+V_{xc}}{2}\right)A_{R}\left(r,\epsilon \right)\right] \end{array}$$

(41) $$\begin{array}{ccc} I & = & 2\int \frac{d\epsilon }{2\pi }\left[f\left(\epsilon +\frac{V+V_{xc}}{2}\right)-f\left(\epsilon -\frac{V+V_{xc}}{2}\right)\right]T\left(\epsilon \right), \end{array}$$

where $A_{R(L)}=\langle r|G\left(\epsilon \right)\Gamma_{R(L)}G^{†}\left(\epsilon \right)|r\rangle$ is the right (left) KS partial spectral function, G(ϵ) the Green’s function of the KS system in the energy representation, and T(ϵ) the transmission function (see Equation (33)). Notice that the right-hand sides are expressed solely in terms of KS quantities, while in the left-hand side we have the many-body quantities. In these expressions, the gate voltage *v* enters in the KS partial spectral function and the transmission coefficient only. We want now to derive an expression for the electrical conductance σ and the Seebeck’s coefficient *S* solely determined from the KS quantities. We are focusing here to the linear-response regime, i.e., we will take at the end the limit V→0. In this limit $V_{xc}\left(n\right)$ vanishes for any density *n*, since otherwise we would have a finite current when no external perturbation is present in contradiction with the theorem of uniqueness. We have

(42) $$\sigma ={\left.\frac{dI}{dV}\right|}_{V=0}=\left(1+{\left.\frac{dV_{xc}}{dV}\right|}_{V=0}\right)\int \frac{d\epsilon }{2\pi }f^{\prime }\left(\epsilon \right)T\left(\epsilon \right)=\left(1+{\left.\frac{dV_{xc}}{dV}\right|}_{V=0}\right)\sigma_{S}$$

where we have introduced the KS conductance $\sigma_{S}=\int d\epsilon /2\pi f^{\prime }\left(\epsilon \right)T\left(\epsilon \right)$. To evaluate the term in the round bracket we use the standard chain rules, remembering that our “variables” are n(r) and *I*,

(43) $${\left.\frac{dV_{xc}}{dV}\right|}_{V=0}=\frac{\partial V_{xc}}{\partial I}\frac{\partial I}{\partial V}+\int dr\frac{\delta V_{xc}}{\delta n(r)}\frac{\partial n(r)}{\partial V}=\frac{\partial V_{xc}}{\partial I}\sigma$$

since the last term vanishes in the linear-response regime, $V_{xc}{\left(n\right)}_{I=0}=0$. Inserting this expansion into the previous result, we find finally

(44) $$\sigma =\frac{\sigma_{S}}{1-\frac{\partial V_{xc}}{\partial I}\sigma_{S}}.$$

Notice that in general we should expect that $\frac{\partial V_{xc}}{\partial I}\neq 0$, and therefore $\sigma \neq \sigma_{S}$. Although for the conductance we can find a general correction, for the Seebeck’s coefficient we can derive an analytical formula only in the Coulomb blockade regime. In this particular physical condition, we are interested only in the total number of particles, $N=\int_{R}drn\left(r\right)$ and we can prove that

(45) $$S=-\frac{\frac{dN}{dT}}{\frac{dN}{d\mu }}$$

if μ is the electrochemical potential. In a similar way as for the conductance, we find for the Seebeck coefficient in the Coulomb blockade regime, the expression

(46) $$S=S_{S}+\frac{\partial v_{Hxc}}{\partial T}$$

where $S_{S}$ is the Seebeck’s coefficient calculated from the Landauer’s approach [73]. We can compare our result with the standard DFT approach and with the exact solution provided by the rate equations [7,74,75]. Other approaches are possible to directly calculate the transmission probability, with and without interaction in the central region [44,76,77]. A direct comparison of the different methods would be desirable, but difficult, since one needs to map each set of parameters appropriately.

**Figure 3:**
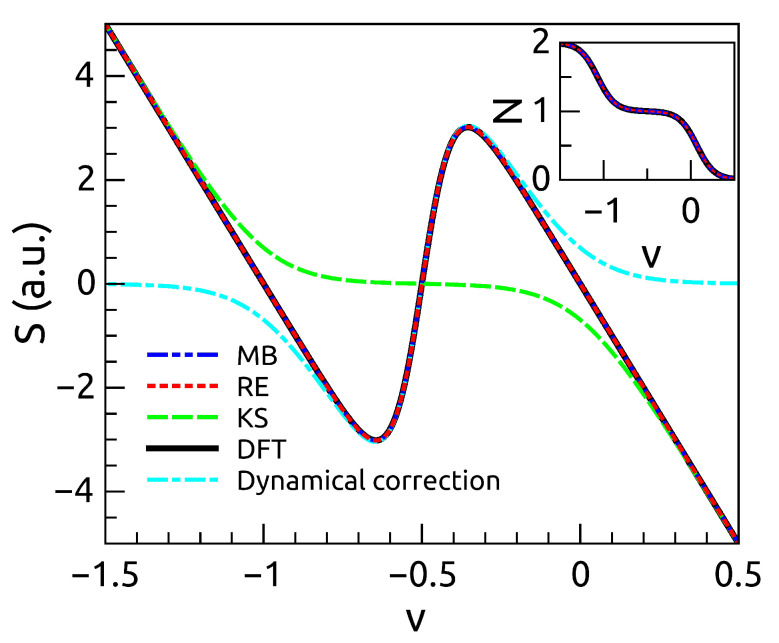
The Seebeck’s coefficient for a single quantum dot in the Coulomb blockade regime as a function of the gate voltage *v*. The exact many-body (MB), the rate equation (RE), and the present theory (DFT) agree quite well in the whole range of the gate voltage *v* considered. The standard KS gives the correct asymptotic but fails in the central region. In cyan, we plot the effect of the dynamical correction $\frac{\partial v_{Hxc}}{\partial T}$. In the inset, we report the total number of particles *N*. All the theories agree quite well for this quantity. Reprinted from [73]. Copyright (2015) American Physical Society.

**Figure 4:**
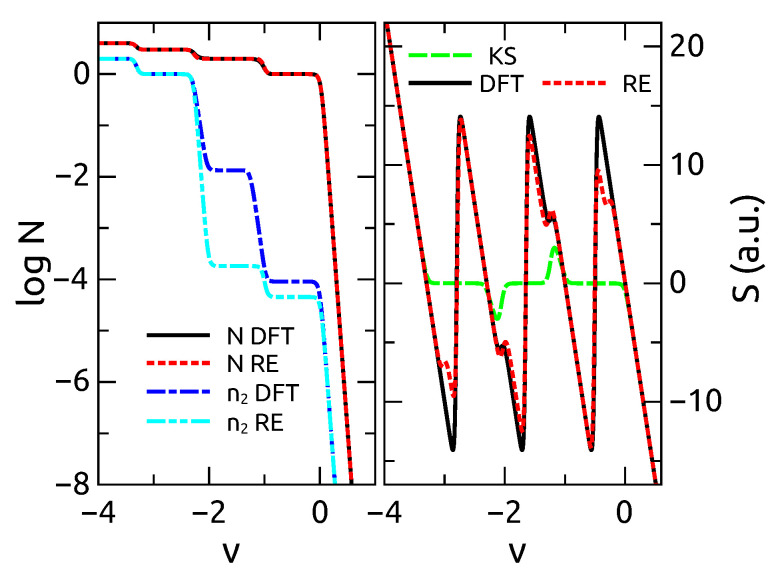
Left pane, the total number of particles *N* and the occupation of the second (highest) energy level as a function of the gate voltage *v*. While the first is exactly reproduced by both DFT and rate equations, the second differs. This originates differences also in the Seebeck’s coefficient which is sensitive to the single particle occupations (right pane). These differences are however small compared with the correction brought about by the dynamical approach. Reprinted from [73]. Copyright (2015) American Physical Society.

Figure 3 report the Seebeck’s coefficient as calculated from the exact many-body theory, the rate equations, the correction Equation (46) and the standard DFT approach $S_{S}$. We notice that the dynamical correction brings $S_{S}$ to coincide with the exact results. Notice that *N* in this case is well reproduced both by the dynamical approach as well as by the standard DFT, therefore in this case is a variable less sensitive to the approximations used. When considering more than one level, we use a constant interaction model. For the case of two levels, we find some discrepancies from the exact theories and the present approach (see Figure 4). This discrepancy originates from using the total number of particles *N* rather than the single occupation of each states $n_{1}$ and $n_{2}$ as our basic variables.

### 3.3. Open-Quantum Systems

The theory of open-quantum systems (OQS) is a well-established model of the coupling between a system and an external environment [78,79,80]. Usually, the latter is considered to be a large reservoir of particles, momentum, and energy which it freely exchanges with the system. The coupling between the system and the environment might be quite general, and we can couple more than one environment, with different macroscopic thermodynamic parameters, such as temperature, chemical potential, or pressure, at a time. Usually the theory is used to describe the dynamical relaxation of the system towards some steady state or thermal equilibrium, but it has found also widespread applications in quantum optics, transport modeling, surface hopping in chemical reactions [81], and so forth (see also Section 3.4).

In the following, we are making some standard assumptions about the environment(s):

(1) They are usually unaffected by the coupling with the systems. This means that the macroscopic parameters that describe the environment are not modified by the coupling with the system. This holds (partially) true if the environment is thought of as made of infinitely many degrees of freedom.
(2) The external parameters are controllable in time, and due to the previous assumption, there is no-feedback between the environment and the system. For example, this implies that the establishment of an electrical current between two reservoirs at different chemical potentials through a molecular junction does not change the electrochemical potential, the temperature, or charge distribution in the reservoirs.

An efficient way to deal with the environment is therefore by tracing out its degrees of freedom and introduce effective correlation functions, (usually dependent on the thermodynamic parameters) with which we describe the dynamics of the quantum system. The way the tracing is made defines the accuracy of the theory. Normally, one can identify two large families of approximations: in the first (called Markovian) the system dynamics does not have any history. The state is determined solely by the evolution at a certain time *t* and does not depend on any time $t^{\prime }<t$. On the other hand, we have non-Markovian dynamics where the state of the system depends on all or some of the previous times, the history of the system, since the initial time $t_{0}$. Into this second family, we then distinguish how the kernel generated by the coupling with the environment depends on the actual state of the system. Let us therefore establish some notation and the standard results. In the following, we will work on a formulation based on the density matrix. Alternative formulations based on a vector state in the Hilbert space are possible and will be briefly discussed later [78,80].

We assume that a system *S* is coupled to an environment *B*. When there is no coupling, the dynamics of *S* is described by a set of operators acting on a Hilbert space $H_{S}$, while the dynamics of *B* by operators acting on $H_{B}$. We further assume that these Hilbert spaces are disjoint, therefore the total Hilbert space of S+B is given by $H_{S}⊗H_{B}$, and thus the state of the total system S+B is represented by a density matrix in this space. It follows that each operator of *S* commutes with each operator of *B*. We now assume that there is a coupling between *S* and *B*, $H_{SB}=\sum_{i}^{q}\lambda_{i}V_{i,S}⊗V_{i,B}$ where $V_{i,S(B)}$ is an operator acting on the $H_{S(B)}$ space, and *q* the number of operators coupling the system with the bath. Notice that this expansion is always possible due to the commutativity between the operators acting on *S* and *B*. For the moment, we focus on the simple case q=1, but the extension to the more general case q>1 is trivial, so in the following we set $\lambda_{i}=\lambda$. The density matrix of the total system evolves according to the von-Neumann equation,

(47) $$\partial_{t}\rho \left(t\right)=-\frac{i}{\hbar }\left[H,\rho (t)\right],$$

where $H=H_{S}+H_{B}+H_{SB}$ is the total Hamiltonian. A basis set for the total Hilbert space is given by {|j,k〉} where *j* is a element of the basis for $H_{S}$ and *k* for $H_{B}$.

Clearly, if we are interested in the dynamics of the system *S* only, this equation contains much more information than needed, since it entails the dynamics of the many degrees of freedom of the environment. We wish therefore to obtain an equation of motion for a density matrix, where the coupling with the environment is effectively described by some macroscopic parameters. This is possible when one assumes that the coupling strength λ is sufficiently small and we can use perturbation theory. The exact definition of the “smallness” of λ is actually an open problem, and we will refer the interested reader elsewhere [78,79,80].

The aim of the theory is to obtain the dynamics of the reduced density matrix, whose element (i,j) is given by

(48) $${\left(\rho_{R}\right)}_{i,j}={\left({\mathrm{tr}}_{B}\rho \right)}_{i,j}=\sum_{k\in H_{B}}\langle i,k|\rho |k,j\rangle .$$

The reduced density matrix is therefore a density matrix in the space of the system *S*, but its dynamics is determined not only by $H_{S}$ but also by the dynamics of the environment. The derivation of the equation of motion for the reduced density matrix of the system stems from the dynamics of the density matrix of the bath when decoupled from the system, $\rho_{B}\left(t\right)=\exp\left(-iH_{B}t\right)\rho_{B}\left(0\right)\exp\left(iH_{B}t\right)$, the factorization of the initial density matrix $\rho \left(0\right)=\rho_{S}\left(0\right)⊗\rho_{B}\left(0\right)$, and the vanishing of the quantum averages of the bath operators $V_{B}$ to first order in λ, ${\mathrm{tr}}_{B}\left(\rho_{B}V_{B}\right)\propto \lambda^{2}$ (this latter assumption can be relaxed and would eventually contribute an effective force that redefines the system Hamiltonian). This standard procedure leads, after some straightforward algebra, to [78,80]

(49) $$\frac{d\rho_{S}}{dt}=-i\left[H_{S},\rho_{S}\right]+\lambda^{2}\left[V_{S},M^{†}\left(t\right)-M\left(t\right)\right],$$

where we have defined

(50) $$M\left(t\right)=\int_{0}^{t}dt^{\prime }C\left(t,t^{\prime }\right)e^{-iH_{S}\left(t-t^{\prime }\right)}V_{S}\rho_{S}\left(t^{\prime }\right)e^{iH_{S}\left(t-t^{\prime }\right)}$$

and

(51) $$C\left(t,t^{\prime }\right)={\mathrm{tr}}_{B}\left[\rho_{B}\left(0\right)V_{B}\left(t\right)V_{B}\left(t^{\prime }\right)\right]$$

is the bath correlation function, where $V_{B}\left(t\right)=\exp\left(iH_{B}t\right)V_{B}\left(0\right)\exp\left(-iH_{B}t\right)$ is the time evolution of the bath operators. Equation (49) is difficult to solve for two reasons. On the one hand, the density matrix is usually a dense matrix of $N^{2}$ elements, if we have *N* elements in the basis set. This usually requires long computation time and large amounts of memory. On the other hand, it contains the full history of the system and at each time step this history needs to be evaluated to calculate the integrals in Equation (50). To deal with the first problem, one can formally derive an equation of motion for a state vector in the Hilbert space that somewhat resembles a wave-function dynamics [82,83,84] and scales as *N* although its physical interpretation is slightly different [85,86]. However, the state vector follows a stochastic dynamic and therefore one needs to average over the realizations of the stochastic noise, balancing the computation gain of the reduced dimensionality. To deal with the second problem, we have two ways: we can neglect completely the history of the system, or retain part of it. In the Markov approximation [78,79,80] one arrives to the so-called Lindblad equation [87]

(52) $$\frac{d\rho_{S}}{dt}=-i\left[H_{S},\rho_{S}\right]+\frac{\lambda^{2}}{2}\left(V_{S}^{†}V_{S}\rho_{S}+\rho_{S}V_{S}^{†}V_{S}-2V_{S}^{†}\rho_{S}V_{S}\right).$$

Lindblad proved that this equation is the most general master equation of the Markov’s type that preserves trace, positiveness and hermiticity of the density matrix $\rho_{S}$ at each time steps up to second order in λ. A second approach follows from the observation that up to second order in λ, $\rho_{S}\left(t\right)=\rho_{S}\left(0\right)$. Then, up to the same order of approximation, one replaces $\rho (t^{\prime })$ with ρ(t), in Equation (50), i.e.,

(53) $$M\left(t\right)\approx \int_{0}^{t}dt^{\prime }C\left(t,t^{\prime }\right)e^{-iH_{S}\left(t-t^{\prime }\right)}V_{S}\rho_{S}\left(t\right)e^{iH_{S}\left(t-t^{\prime }\right)}.$$

This operator is now local in time, although one needs to calculate it at each time step. In the absence of any magnetic field, the bath dynamics satisfies time-reversal symmetry, and this is usually sufficient for $C\left(t,t^{\prime }\right)=C\left(t-t^{\prime }\right)$, especially if $\rho_{B}\left(0\right)$ is the equilibrium statistical density matrix of the bath $\rho_{B,equ}=\exp\left(-\beta H_{B}\right)$ where $\beta =1/k_{B}T$ and *T* the bath temperature, we can separate this integral and arrive at the Redfield master equation [80,88].

To describe thermal transport within OQS one needs to couple two environments, kept at different temperature, locally to the system

(54) $$\frac{d\rho_{S}}{dt}=-i\left[H_{S},\rho_{S}\right]+L_{L}\left[\rho_{S}\right]+L_{R}\left[\rho_{S}\right].$$

**Figure 5:**
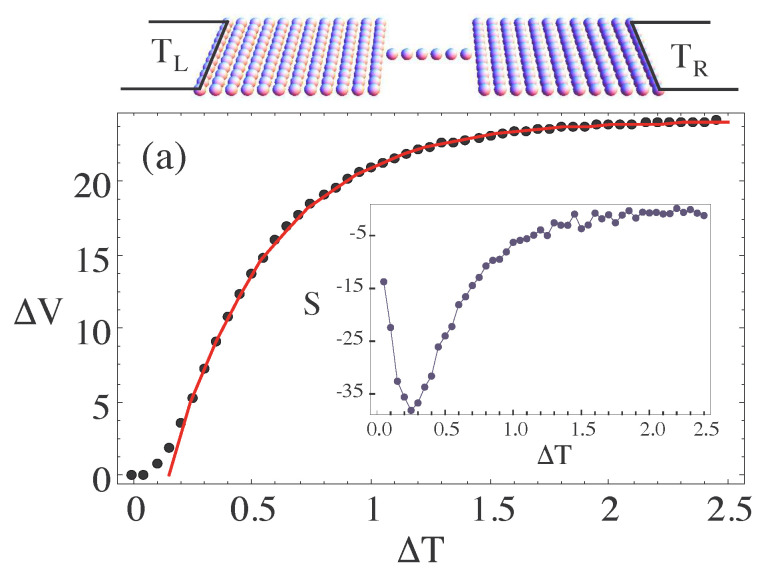
Upper Panel: Nanostructure connected locally to two reservoirs kept at different temperature. Below: Voltage drop, ΔV as a function of the temperature gradient, ΔT. The inset shows the thermopower $S=-\frac{d(\Delta V)}{d(\Delta T)}$. Reprinted (adapted) with permission from [89]. Copyright (2009) American Chemical Society.

Here, $L_{L}$ and $L_{R}$ describe the left and right environment, respectively. A possible nano-junction attached to two environments is shown in the upper panel of Figure 5. Furthermore, in Figure 5a one sees the voltage drop over the device induced by the applied temperature gradient, ΔT, due to the environmental coupling. One can observe a linear-response regime for small ΔT, a regime of rapid rise in ΔV and for large temperature gradients a region where the voltage drop has reached saturation due to the finite size of the system. The inset of Figure 5 shows the thermopower S=−d(ΔV)/d(ΔT) that presents a maximum in response to the thermal gradient at ΔT≈0.25 [a.u.].

Note that all the shown quantities correspond to the steady-state solution of the master equations, where the long-time limit has been reached. In general, the OQS approach is not limited to this regime and can also be used to study time-dependent phenomena in nanoscale devices beyond linear response [78,90,91].

For the purposes of this review, it is relevant to point out that the quantum system is in general a device made of interacting electrons and ions. This, as we have seen (Section 3.1.1), is an incredibly complex problem and the coupling with the external environment does not make it simpler. Fortunately, we can use the theory of open quantum systems with (time-dependent) DFT to extract from a non-interacting open-quantum system information about the dynamics of the interacting one [58,83,92,93,94,95]. More surprisingly, it has been shown that the DFT for open-quantum system can be constructed in such a way that the KS system can be made closed. This means that the effects of the external environment can be included in the KS potentials [94,95]. However, it is not clear how one could effectively build the required KS potentials which would be depending on the coupling operators between the system and the environment. These difficulties have so far hindered a full development of the theory and its routine application for the investigation of transport in complex devices and physical conditions.

### 3.4. Influence of Decoherence onto Thermoelectrical Transport

The observation or measurement process acting on a classical object does not influence its physical properties. However, when entering the nanoscale world and the quantum realm one needs to revisit some classical concepts. In the smallest nanodevices, electrons are usually set up in coherent superpositions, and a global measurement would destroy most of their coherence changing the dynamical response. Decoherence does not only originate from the measurement by a macroscopic observer, but it also occurs when the quantum system interacts locally with another quantum system. This effect of local quantum observation covers a variety of situations, such as, e.g., local electron–phonon coupling and continuous [96] or frequent [97] quantum non-demolition measurements. It has been shown that decoherence influences the efficiency of energy transport in biological [98,99,100] and molecular devices [100,101,102] and is responsible for the decoupling of the system from the environment via the Quantum Zeno effect [103,104].

While standard static transport approaches such as the BTE or LB fails in catching up with these dynamical effects, methods such as TDDFT or OQS approaches allow for the time-resolved study of electron transport and energy dissipation. Additionally, methods describing the dynamics within a density matrix formalism are suitable to study the role of coherence in nanoscale transport devices. Indeed, by modeling decoherence as an additional environment within a master equation approach, one can include it into a consistent thermodynamic formalism [105],

(55) $$L_{D}\left[\rho_{S}\right]=\lambda_{D}\left[2|\beta \rangle \langle \beta |\rho_{S}\left|\beta \rangle \langle \beta \right|-\left|\beta \rangle \langle \beta \right|\rho_{S}-\rho_{S}\left|\beta \rangle \langle \beta \right|\right].$$

**Figure 6:**
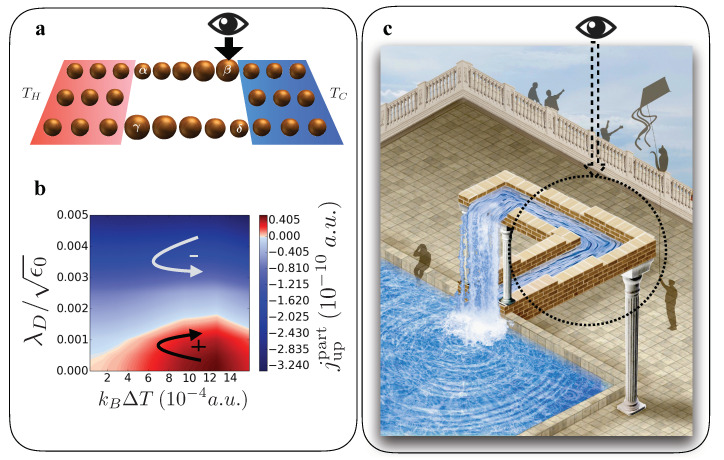
Influence of quantum decoherence on the thermoelectric flows in a quantum ratchet shown in (**a**). (**b**) The electrical current in the upper branch can be seen. The current can change direction as a function of both $\lambda_{D}$ and ΔT. (**c**) Artistic illustration (copyright K. Aranburu) of the role of decoherence in a nanodevice: When observing inside the black circle, it appears the water flows down the channel, instead, by looking at the whole painting the water actually flows uphill. This apparent paradox mimics the coherent superposition of two quantum states (water flowing up/down). By observing at specific parts of our system one is able to tune between these two states and hence change the ‘physical response of the nanodevice’ in a controlled way. Parts (**a**,**b**) are adapted from [105]. Copyright (2017) SpringerNature.

Here |β〉 is the ket-vector representing the state of the spatial region where decoherence takes place. This bath can change the coherence of the system and has; in contrast to classical reservoirs that are in a thermal ensemble state, no temperature is associated with it [106]. This formalism has been applied to a ratchet-like device shown in Figure 6a [105].

By changing the on-site energy levels on the parallel horizontal branches of the device (graphically indicated by the size of the spheres), a spatial asymmetry is introduced. In the upper branch the on-site energies increase from left to right in equal proportion, while in the lower branch this is reversed. Therefore, the device acts as a quantum ratchet by the presence of two rectifiers on each branch in opposite direction. This directional transport device driven by thermal and quantum fluctuations has a preferred electronic current direction, in this example clockwise. In the ratchet, a hot (*H*) and cold (*C*) reservoir introduce a thermal gradient in the device while at the same time the decoherence bath (*D*) is acting at site β

(56) $$\frac{d\rho_{S}}{dt}=-i\left[H_{S},\rho_{S}\right]+L_{H}\left[\rho_{S}\right]+L_{C}\left[\rho_{S}\right]+L_{D}\left[\rho_{S}\right].$$

While in this set-up energy is exchanged via the baths, particle current is then confined inside the device. Figure 6b shows the electronic current at steady state for the upper branch. Please note that by charge conservation, the current in the lower branch is exactly the same but flowing in the opposite direction. Here, red indicates a positive current, from left to right in the upper branch. If no decoherence is applied ($\lambda_{D}=0$), the current is flowing in the clockwise direction through the ratchet. Increasing the decoherence, the ring current decreases until its direction gets reversed. It has been found that the local quantum observer cannot only control the particle current but also energy currents in direction and strength inside the device [105]. This demonstrates that in thermoelectric nanodevices the current and heat flows are not only dictated by the temperature and potential gradients, but can also be manipulated by the external control of the coherence of the device. This effect is illustrated in Figure 6c. When looking at the whole picture the water flows uphill, however, when we only observe current flow in the circle (and ignore the rest of the illustration) it seems the water flows down the channel. This apparent paradox mimics the coherent superposition of two quantum states (water flowing up/down). The observation process in specific parts of our system can tune between these two quantum states and hence change the ‘physical response of the nanodevice’ in a controlled way.

### 3.5. Time-Dependent Thermal Transport Theory

Modeling thermal transport and thermoelectric effects at the nanoscale is difficult, because besides the intricacies of certain materials, we lack a working definition of temperature at those scales. Indeed, the concepts of equilibrium and thermodynamic limits are difficult to extend to the case of a few electrons or phonons strongly localized in a nanodevice. Clearly, also the idea that the leads/reservoirs have a well-established temperature (distribution function) down to the contact with the device is an abstraction. Many attempts have been tried to remedy this situation and general extensions of some thermodynamics concepts to the nanoscale have been attempted [107,108].

One of such attempts is to completely remove the needs for macroscopic reservoirs and leads by replacing them with an effective radiation of known properties. One might think for example, of replacing the electrostatic bias with the time-dependent vector potential that produces the same electric field. A similar attempt can be made with the temperature gradient. We replace the coupling to the energy reservoirs by two black bodies each radiating according to their own temperature and spectral properties: the electromagnetic field of the blackbody radiation is then included in the system dynamics and establish in this way the needed temperature gradient and transport dynamics. To maintain a steady state and avoid that the energy stored in the device saturates, we assume that the system is weakly coupled to an external environment at a given temperature. This corresponds to an experimental set-up; the quantum systems gets excited out of its equilibrium by thermal radiation and at the same time can release energy to its surrounding. (such as a metal heated up by a laser and starts to glow). Recent progress has been made to include these quantum-electro-dynamical effects into an ab-initio formalism [109]. Being able to study and detect the radiation produced by the transport dynamics could possibly allow the definition of an effective “local temperature” based on the electrodynamics theory.

**Figure 7:**
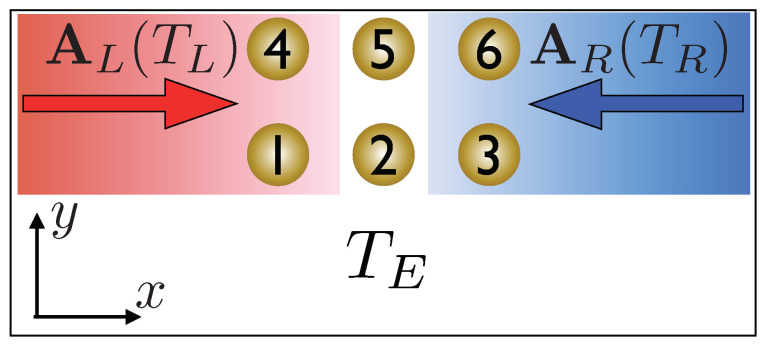
Set-up under consideration. Six tight-binding sites are connected locally to the radiation of two blackbody at temperature, $T_{L}$ and $T_{R}$. Furthermore, the system is connected to an environment at temperature $T_{E}$. Reprinted (adapted) from [109]. Copyright (2015) American Physical Society.

We assume each blackbody to be located far away from the devices, therefore for all our purposes we will consider the radiation they produce as composed solely of plane waves propagating along the line connecting the device and the blackbody. In Figure 7 the considered nanodevice is shown and the left and right blackbody radiation is indicated by $A_{L}$ and $A_{R}$ respectively. While the blackbodies radiate according to their respective temperature $T_{L}$ and $T_{R}$, the system is interacting with an environment at temperature $T_{E}$. The amplitude of each plane wave of the blackbody field is determined by the temperature of the blackbody itself. We assume therefore the thermal radiation of the form

(57) $$A_{L(R)}\left(r,t\right)=E_{0}\int d\Omega \sqrt{\Omega n\left(\Omega ,T_{L(R)}\right)}\sin\left(kx\pm \Omega t+\varphi \left(\Omega \right)\right),$$

where n(Ω,T) is the Bose-Einstein distribution, $E_{0}=E_{0}e_{y}$. Here, $E_{0}$ is the strength of the corresponding electric field, where $e_{y}$ is the unit vector in the y direction and *k* is the momentum of the photon with frequency Ω. We have considered a random phase, φ(Ω)∈[0,2π) that ensures the plane waves are incoherently reaching the device. In principle, there are more realistic, but numerically more expensive, ways to model the blackbody thermal radiation [110,111]. A comparison of these methods with the results of Equation (57) is still missing. Due to the coupling with an external environment, we expect that the system reaches a steady state. This coupling is effectively described through a master equation where the environmental power spectrum for $|\omega |<\omega_{c}$ is

(58) $$C\left(\omega \right)={4|\omega |}^{3}n(|\omega |,T_{E})+\theta \left(-\omega \right),$$

where θ(ω) is the Heaviside step function, and $\omega_{c}$ is a cutoff frequency determined by the scale of the system. For $|\omega |>\omega_{c}$, C(ω)=0. We apply this model to the case of a device described in the tight-binding formalism where the electrons are not interacting. In this case, the Hamiltonian for the system is given by

(59) $$H_{s}=\sum_{\langle i,j\rangle }T_{i,j}c_{i}^{†}c_{j},$$

where the operator $c_{j}$ destroys an electron in the site *j* and $T_{i,j}$ is the hopping parameters. The blackbody radiation enters here as a Peierls transformation into the Hamiltonian

(60) $$T_{i,j}=t_{i,j}\exp\left[-i\int_{R_{i}}^{R_{j}}dr\left(A_{L}\left(r,t\right)+A_{R}\left(r,t\right)\right)\right],$$

where $t_{i,j}$ is the hopping parameter in the absence of the radiation. We align the device along the direction joining the two blackbodies. If one considers initially the case where the blackbodies and the environment have the same temperature, we see that the dynamics of the system points towards a steady state with no net energy transport in the long-time limit [109].

**Figure 8:**
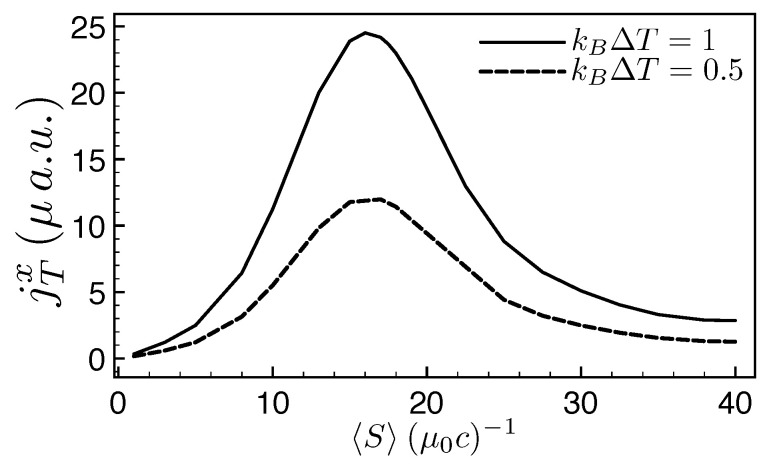
Thermal current along the *x* direction for different temperature gradients as a function of the strength of the coupling 〈S〉. For large coupling, the current decreases since the energy level are all filled, and the dynamics is quenched. Reprinted (adapted) [109]. Copyright (2015) American Physical Society.

In the case of a finite temperature gradient we observe in Figure 8 a turnover of the energy current, $j_{T}$, with respect to the flux of energy from the blackbodies. Here, 〈S〉 is the time-averaged Poynting vector indicating the coupling strength to the blackbodies. Indeed, for small fluxes, the energy current increases when increasing the flux, then reaches a maximum and later vanishes for intense radiations. The reason for this turnover lies in the finite number of states available to carry energy. For small fluxes almost all states are empty and there is the possibility to allocate for a larger number of carriers. When the flux increases, the states fill up, and less and less will be available for conduction, until essentially all the states will by full and no dynamics is possible [109]. We want to point out that this theory can be applied to large temperature gradients: the coupling with the black body radiation is not restricted to linear response, while the system is coupled weakly to the environment [109].

## 4. Discussion

The investigation of energy and electrical transport in nanoscale devices has recently attracted a consistent amount of interest for its wide range of potential applications in technology, biomedicine and energy materials to name just a few. A comprehensive look at the field would, therefore, require more than a reasonably short review. We have preferred a bird-eye introduction to what we believe are the actual more promising theoretical methods, the Boltzmann’s equation and the Landauer-Büttiker formalism for quantum transport. These methods are somewhat complementary since they apply to different regimes, namely a diffusive regime where scattering events are important, or a ballistic regime where the quantum-mechanical effects are dominant. While these methods might not be the most accurate for any situation, most of the recent progress in our understanding of thermal, energy, and electronic transport at the nanoscale is based upon them or some of their extensions. Both methods benefit from an accurate description of the quantum-mechanical properties of the material under investigation. In this respect, the method of choice is Density Functional Theory, since its practical implementation allow for an almost straightforward interfacing with both the Boltzmann or Landauer theories (the last one through the Non-Equilibrium Green’s functions formalism). Therefore, the use of DFT methods for electronic structure calculations (including phonon and vibration properties) combined with Boltzmann’s or Landauer’s theories is to some extent the state of the art of modern calculations.

We have briefly discussed the need to go beyond this *state of the art* both in terms of the limits of DFT or the limits of the approximations used in the methods themselves. Clearly, this is a very fertile field of investigation and in most cases it requires novel paradigms. We have exposed a few here: the introduction of methods beyond the constant relaxation time approximations ameliorates the description of electron and phonon transport by introducing energy-dependent relaxation times; The static DFT cannot, in principle, be the theory to study transport, an inherent out-of-equilibrium problem. We therefore introduce extension of DFT able to properly take into account the dynamics of the electrons and show how this theory naturally allows for the description of strongly correlated effect in nanoscale molecular transport, in particular how it corrects the calculation of the Seebeck coefficient. We finally introduced the theory of open-quantum system, where the system under investigation is “open” to an external environment with which it could exchange energy, momentum, and particles. The external environment might have a passive role of driving an otherwise out-of-equilibrium system toward a steady or ground state, or an active role in which it drives the system itself allowing for novel quantum-mechanical effects to be exploited. As the OQS approach describes the dynamics of the density operator, this theory can be also used to investigate the influence of coherence in quantum transport devices. This might pave the way for novel strategies to construct quantum devices for thermoelectric energy conversion, photonics, spintronic injection, and sensing, to just name a few.

Clearly, the previous description cannot be complete. For example, we are aware of other DFT methods to include thermoelectric phenomena [64,65] or strong correlations [52,112,113,114] in the Kohn-Sham system, or an accurate description of vibrational modes and energy transport with classical methods by solving the Newton’s equations [115,116], or the inclusion of non-harmonic effects in the non-equilibrium Green’s function for the phonons [117]. Each of these topics deserves a complete introduction per se.

We hope this review serves both to the young researcher to have an outlook of the actual state of the art and potential ways beyond it, without striving through the immense amount of literature currently available and in continuous development, and to the experienced scientist as a reference where some of the most fundamental results and future outlooks are collected.

## Abbreviations

The following abbreviations are used in this manuscript:

ALDA	Adiabatic Local Density Approximation
BTE	Boltzmann Transport Equation
LBTE	Linearized Boltzmann Transport Equation
CRTA	Constant Relaxation Time Approximation
DFT	Density Functional Theory
EPA	Electron–Phonon Averaged approximation
KS	Kohn–Sham
LB	Landauer–Büttiker
NEGF	Non-Equilibrium Green Function
RG	Runge–Gross
TDDFT	Time-Dependent Density Functional Theory
TDCDFT	Time-Dependent Current-Density Functional Theory
OQS	Open-Quantum Systems
SERTA	Self-Energy Relaxation Time Approximation
EPW	Electron–Phonon Wannier
